# Considerations for the Terminal Sterilization of Oligonucleotide Drug Products

**DOI:** 10.1089/nat.2022.0073

**Published:** 2023-06-02

**Authors:** Daniel Paul DeCollibus, Justin Searcy, Anna Tivesten, Nadim Akhtar, Christian Lindenberg, Nounja Abarrou, Sujana Pradhan, Maggie Fiandaca, Jenny Franklin, Geetha Govindan, Hung-Yi Liu, David Royle, Patrick Lim Soo, Kirsten Storch

**Affiliations:** ^1^ Drug Product Technology and Engineering, Amgen, Cambridge, Massachusetts, USA.; ^2^ Pharmaceutical Development, Ionis Pharmaceuticals, Inc., Carlsbad, California, USA.; ^3^ CVRM CMC Projects, Pharmaceutical Sciences, AstraZeneca R&D, Gothenburg, Sweden.; ^4^ New Modalities and Parenteral Development, Pharmaceutical Technology & Development, Operations, AstraZeneca, Macclesfield, United Kingdom.; ^5^ Global Drug Development, Technical Research & Development, Novartis Pharma AG, Basel, Switzerland.; ^6^ GSK, Strategic External Development, Analytical Development, Collegeville, Pennsylvania, USA.; ^7^ CMC Regulatory Affairs, Ionis Pharmaceuticals, Inc., Carlsbad, California, USA.; ^8^ Pharmaceutical Operations & Technology, Biogen, Cambridge, Massachusetts, USA.; ^9^ Pharmaceutical Research and Development, BioTherapeutics Pharmaceutical Sciences, Pfizer, Andover, Massachusetts, USA.; ^10^ Pharma Technical Development, Roche Diagnostics GmbH, Mannheim, Germany.

**Keywords:** terminal sterilization, oligonucleotide, drug product, autoclave, degradation, formulation

## Abstract

A primary function of the parenteral drug product manufacturing process is to ensure sterility of the final product. The two most common methods for sterilizing parenteral drug products are terminal sterilization (TS), whereby the drug product is sterilized in the final container following filling and finish, and membrane sterilization, whereby the product stream is sterilized by membrane filtration and filled into presterilized containers in an aseptic processing environment. Although TS provides greater sterility assurance than membrane sterilization and aseptic processing, not all drug products are amenable to TS processes, which typically involve heat treatment or exposure to ionizing radiation. Oligonucleotides represent an emerging class of therapeutics with great potential for treating a broad range of indications, including previously undruggable targets. Owing to their size, structural complexity, and relative lack of governing regulations, several challenges in drug development are unique to oligonucleotides. This exceptionality justifies a focused assessment of traditional chemistry, manufacturing, and control strategies before their adoption. In this article, we review the current state of sterile oligonucleotide drug product processing, highlight the key aspects to consider when assessing options for product sterilization, and provide recommendations to aid in the successful evaluation and development of TS processes. We also explore current regulatory expectations and provide our interpretation as it pertains to oligonucleotide drug products.

## Preamble

The European Pharma Oligonucleotide Consortium (EPOC) [1] is a collaboration between multiple pharma companies with the aim of sharing chemistry, manufacturing, and control knowledge, as well as strategies to enable harmonization of oligonucleotide development and commercialization. The objective of the consortium is to publish science-based recommendations for the development of oligonucleotide therapeutics in a series of technical and regulatory white papers, drawing on its collective subject matter expertise and complementing that in the literature and guidelines. This public body of prior knowledge endeavors to serve as a reference for industry practice and help establish development principles for oligonucleotides. The consortium aims at being proactive and inclusive, and it anticipates initiating wider discussion on oligonucleotide CMC practice and policy, thus expediting access to these potentially life-changing medicines.

In this article, the authors attempt to provide scientific advice on the topic of terminal sterilization of oligonucleotide drug products. The reader is cautioned that adherence to the advice given in the following sections does not guarantee regulatory agency endorsement of the approaches described. For this reason, sponsors of oligonucleotide therapeutics are strongly encouraged to discuss all questions pertaining to the sterilization process selection with regulatory agencies during the drug development process.

## Introduction

### Oligonucleotides

Oligonucleotides represent a broad spectrum of modified nucleic acid therapeutics. The mechanism of action for most oligonucleotides is derived from Watson-Crick base pairing with target DNA or RNA sequences causing an upregulation or downregulation of protein synthesis, impacting a particular disease pathway. Since the potential use of oligonucleotides as therapeutics was first recognized in 1978 [2], many modifications to the oligonucleotide backbone, sugar, and nucleobase have been developed to increase potency, half-life, stability, and selectivity. Single-stranded antisense oligonucleotides (ASOs) were the first oligonucleotides to be used in medicine and represent most marketed oligonucleotide therapeutics, although increasing numbers of double-stranded oligonucleotides exploiting RNA interference mechanisms (siRNA), oligonucleotides conjugated to targeting moieties, highly modified ASOs (eg, peptide nucleic acids, morpholino), and oligonucleotide aptamers are also being developed.

As a result of their efficacy, selectivity, and capability of treating a large number of diseases and accessing previously undruggable targets, oligonucleotides have rapidly emerged as a major class of therapeutics. Currently, there are at least 14 oligonucleotide products marketed globally and more than 200 in various phases of development. These numbers are expected to grow in the coming years as the utility of oligonucleotides for treating both rare and high prevalence diseases, such as cancers, continues to be realized.

All marketed and most investigational oligonucleotide products in clinical development are delivered as sterile preparations for parenteral delivery. This includes, but is not limited to subcutaneous, intravenous, intramuscular, intravitreal, and intrathecal administration. All marketed oligonucleotide drug products utilize glass prefilled syringes (PFS) or glass vials as their primary packaging (Table 1).

**Table 1.** Marketed Injectable Oligonucleotide Products and Publicly Available Information on Packaging and Sterilization Method (from European Public Assessment Reports)

INN	Chemistry	Company	Primary container	Drug product sterilization process	Summary of data on TS provided in dossier
Volanesorsen	ASO	Ionis	Prefilled syringe	Sterile filtration	Robust steam sterilization process was shown to cause significant impact on product quality. [EMA/180717/2019]
Inotersen	ASO	Ionis	Prefilled syringe	Sterile filtration	Steam sterilization for 8 min at 121°C was shown to cause significant impact to product quality. [EMA/411876/2018]
Nusinersen	ASO	Biogen	Glass vial	Sterile filtration	Sterile filtration and aseptic processing were justified in line with the decision trees for the selection of sterilization methods. [EMA/289068/2017]
Patisiran	siRNA (LNP formulation)	Alnylam	Glass vial	Sterile filtration	Sterile filtration and aseptic processing were justified by the applicant. [EMA/554262/2018]
Givosiran	siRNA	Alnylam	Glass vial	Sterile filtration	Dry heat, steam sterilization, and gamma irradiation were shown to cause significant impact to product quality. Temperatures required for terminal sterilization exceed the melting temperature of siRNA duplex. [EMA/CHMP/70703/2020]
Mipomersen	ASO	Genzyme	Glass vial	Sterile filtration	Steam sterilization for 15 min at 121°C was shown to cause significant impact on product quality. [EMA/305826/2013]
Defibrotide	Mixture of ssDNA and dsDNA	Gentium	Glass vial	Sterile filtration	No detail provided in public assessment report.
Viltolarsen	ASO	NS Pharma	Glass vial	n.a.^a^	No public assessment report available.
Eteplirsen	ASO	AVI Biopharma	Glass vial	Sterile filtration	A feasibility study to evaluate the impact of terminal sterilization by heat on the finished product was conducted. The results of the study demonstrate that terminal sterilization leads to unacceptable product degradation and, therefore, the practice of sterile filtration (using a bacterial-retentive filter membrane) with aseptic processing is the method of choice for the sterile manufacture of the finished product. [EMA/691796/2018]
Golodirsen	ASO	Sarepta Therapeutics	Glass vial	n.a.^a^	No public assessment report available.
Inclisiran	siRNA	Novartis	Prefilled syringe	Sterile filtration	Sterile filtration and aseptic processing were justified. The use of heat, gamma irradiation, or chemical sterilization methods present unacceptable risk to product quality due to the nature of the active substance. [EMA/696912/2020]
Fomivirsen	ASO	Ionis Novartis	Glass vial	Sterile filtration	No public assessment report available.
Pegaptanib	Aptamer	NeXstar PharmaEyetech Pharma	Prefilled syringe	n.a.^a^	No detail provided in EMA public assessment report.
Lumasiran	siRNA	Alnylam	Glass vial	Sterile filtration	The use of sterile filtration and aseptic processing were justified with literature references showing significant impacts on product quality are expected for the class of molecule. The temperatures required for thermal sterilization exceed the melting temperature of siRNA duplex. [EMA/568312/2020]
Casimersen	ASO	Sarepta Therapeutics	Glass vial	n.a.^a^	No public assessment report available.
Vutrisiran	siRNA	Alnylam	Prefilled syringe	Sterile filtration	Steam sterilization of the finished product is not feasible since the melting temperature of the duplex structure is at 83°C. [EMA/CHMP/689555/2022]

^a^ Not available [no public information in EMA public assessment report or FDA product quality review(s) ]

ASO, antisense oligonucleotide; EMA, European Medicines Agency; TS, terminal sterilization.

A survey performed of EPOC partner organizations also revealed that oligonucleotide drug product presentations in development for parenteral delivery are also limited to stand-alone glass PFS, glass PFS integrated into an autoinjector device, and glass vials. In addition, the member organizations indicated most products are formulated in water, simple aqueous buffer matrices, for example, phosphate-buffered saline, or fit-for-purpose aqueous buffers such as artificial cerebrospinal fluid, which is used for intrathecal delivery of SPINRAZA^®^. Only 1 of 8 member organizations surveyed was currently developing formulations using stabilizing excipients, and the same number was exploring advanced formulations for enhanced drug delivery, such as nanoparticles.

### Sterile drug product manufacturing

Manufacturing of oligonucleotides typically begins with the synthesis of the oligonucleotide sequence by sequential coupling of starting materials (phosphoramidites). The crude material is cleaved from the solid support and, before or after a conjugation step in case of conjugated oligonucleotides, chromatographically purified to yield a nonsterile, low bioburden drug substance. In most cases, the drug substance is a lyophilized powder of the active pharmaceutical ingredient (API), although processes yielding drug substance in aqueous solution are also being utilized [3].

The drug product process generally consists of a dissolution and/or formulation step, sterilization by membrane filtration, and aseptic filling and finish. For oligonucleotide products with advanced drug delivery formulations, for example, lipid nanoparticles, additional processing steps are typically required (eg, homogenization). The sterilization technique used for currently marketed oligonucleotide drug products, for which public information is available, is exclusively by membrane filtration. Membrane filtration is suitable for oligonucleotides as their formulations are compatible with common sterilizing grade membranes, the manufacturing processes are well established and characterized, and most manufacturers have internal manufacturing capacity or access to contract manufacturers with aseptic manufacturing capabilities. In most cases, the justification for using sterile filtration and aseptic processing over terminal sterilization (TS) was due to an observed negative impact of TS to product quality (Table 1).

## Problem Statement

It is a clear regulatory expectation that TS is the preferred method for sterilizing parenteral products [4–6]; however, it is less clear the calculus that goes into determining if TS is feasible for a given product. This is partly due to the lack of a sufficiently detailed and internationally harmonized guidance. The latest regional guidance on the topic, the 2019 European Medicines Agency (EMA) Guideline on the Sterilisation of the Medicinal Product, Excipient and Primary Container [4], provides some direction through the inclusion of decision trees for choosing the appropriate sterilization methods, suggestion of minimum sterilization requirements, and defining the information to be submitted in the marketing application, which includes a risk-benefit analysis if aseptic processing is to be used [4].

The guidance also recommends that “substantial efforts should be made to enable TS,” including the exploration of formulations, container closures, and process parameters that may increase the chance of successful implementation of TS processes. Although the EMA guidance provides greater insight into the regulator's perspective and covers concepts that were previously unaddressed, there remains uncertainty regarding how to put this guidance into practice. There also remains ambiguity around subjective language used in the guidance, such as “substantial efforts,” or what is considered acceptable risk.

A chief concern for drug developers is balancing the risk of negatively impacting product quality during TS against the perceived risks of aseptic processing, that is, release of a nonsterile unit. For sensitive products, such as biologics, doublestranded oligonucleotides, and products requiring advanced formulations for delivery, for example, nanoparticles, the scale is heavily tipped toward using membrane filtration and aseptic processing. This is because the stresses associated with TS are likely to be detrimental to product quality, and such impacts can be readily demonstrated during development. However, for more stable molecules, it is possible that only moderate changes in the impurity profile may occur, making the decision of which sterilization method to use more difficult and opening the door for differing opinions between manufacturers and regulators.

There are many technical considerations that add to the complexity, such as identifying formulations and container closures amenable to TS, choosing the right sterilization equipment and conditions, ensuring the process is robust and can be validated, and ensuring the shelf life of the product is not compromised. Furthermore, the reliance on platforms and similarities in ASO formulations, manufacturing processes, impurities, and toxicology profiles [7] add weight to each decision, as each may set precedent within a manufacturer's development pipeline and across the industry. Therefore, a unified understanding of the subject matter is needed and EPOC is uniquely positioned to help communicate these considerations on behalf of oligonucleotide manufacturers.

## Scope of the Article

In the following sections, we provide our interpretation of the current regulatory guidance for commercial process development and highlight key concepts regarding the TS of oligonucleotides. Included are recommendations for formulation development, assessing changes in the purity and impurity profile after TS, and selecting the correct container closure. We also touch on requirements for autoclave equipment capabilities and considerations for autoclave cycle development. Based on the current oligonucleotide development landscape and exclusion of oligonucleotide subclasses that are unlikely to have success with TS (eg, siRNA), a particular focus is given to single-stranded ASOs in aqueous formulations in glass primary containers, such as vials and PFS. Accordingly, the use of ionizing radiation and chemical sterilization methods is excluded from the discussion as they are not compatible with aqueous formulations of oligonucleotides [8], which is in alignment with the decision tree for aqueous products in the EMA Guidance.

## Regulatory Overview

Regulatory guidance specific to oligonucleotides is limited. Oligonucleotide manufacturing control strategies are often derived from a mixture of guidance documents related to both synthetics and biologics; however, oligonucleotides are ultimately reviewed as synthetic drugs. For example, oligonucleotides are fully or partly out of scope of several of the current ICH guidelines, such as Q3A, Q3B, Q6A, and M7. There are areas where the traditional small molecule principles can easily be applied, such as for solvents, elemental impurities, and small molecule impurities that may be present in the drug substance. For other areas, it is not as straightforward to adapt small molecule requirements, as is the case for oligonucleotide-related impurities [7].

When selecting a sterilization method, limited guidance relevant or specific to oligonucleotides is available. Table 2 gives an overview of available guidance related to sterilization, including high-level information of scope and recommendations given. Most available guidance describes detailed requirements for facilities, quality systems, process validation, and regulatory documentation, but do not provide any criterion for the selection of a sterilization process. This gap in regulatory guidance was substantially filled in 2019 when the EMA Guideline on the “Sterilisation of the medicinal product, active substance, excipient and primary container” was made effective [4]. As the most prescriptive guidance on the topic in most areas, it sets the bar for TS process development. As such, a focused review and assessment of its contents, as related to manufacture of oligonucleotides, is provided in the following section.

**Table 2.** Overview of Guidance's Covering Sterilization of Aqueous Drug Products [4–6,30,31]

	EMA 2019 [4]	PMDA 2012 [5]	FDA 2010 [6]	FDA 2004 [30]	FDA 1994 [31]
Selection of sterilization method	Yes – decision tree (steam sterilization for aqueous products)	No	No	No	No
Validation of process	Yes (general principles only)	Yes	No	Yes	Yes
Data in quality dossier	Yes	Yes	Yes	No	Yes
Quality system & manufacturing facility controls	No	Yes	No	Yes	No
Preferred conditions for terminal sterilization by steam	≥121°C for ≥15 min (Reference conditions of Ph. Eur. 5.1.1)	≥121.1°C for ≥15 min	N/A	N/A	N/A
Minimum conditions for terminal sterilization by steam	F_0_ ≥8 min with hold temperature ≥110°C AND achieving SAL ≤10^−6^	F_0_ ≥8 min OR alternative conditions achieving SAL ≤10^−6^	N/A	N/A	Conditions achieving SAL ≤10^−6^
Product quality attribute criteria beyond sterility	“If impurities are either metabolites or are generated at levels already qualified, then terminal sterilization is still considered feasible. However, if the degradation products are not qualified at the level at which they occur, then sterile filtration and aseptic processing may be selected.” Other CQA:s not discussed	Not in scope beyond mentioning that product requirements after the sterilization process should be specified	Not in scope	Not in scope	Not in scope beyond mentioning “The program for monitoring the stability of the packaging and integrity of the container-closure system barrier over the claimed shelf life should be described”
Selected take home messages	“Terminal sterilization provides the highest assurance of sterility and should be used whenever possible.” “[...] substantial efforts should be made to enable terminal sterilization.” “For highly sensitive products, such as most biologic products, where terminal sterilization of the finished product is not acceptable, sterile filtration and/or aseptic processing under validated and controlled conditions can be accepted.” “The acceptability of aseptic processing should be based on the application of the decision tree and a risk assessment.” Data and justification are required for any option not meeting Ph. Eur. 5.1.1.	“This guidance is applicable to the quality system governing all processes in manufacturing sterile pharmaceutical products at facilities where products are manufactured by terminal sterilization procedures.”	“This guidance provides recommendations to applicants on information to include in support of parametric release for sterile products terminally sterilized by moist when submitting a new drug application (NDA), abbreviated new drug application (ANDA), abbreviated new animal drug application (ANADA), biologic license application (BLA), or supplement or other postmarketing report” “The principles of this guidance may also be applicable to products sterilized by other terminal sterilization processes.”	“This guidance is intended to help manufacturers meet the requirements in the Agency's current good manufacturing practice (cGMP) regulations (21 CFR parts 210 and 211) when manufacturing sterile drug and biological products using aseptic processing” “It is a well-accepted principle that sterile drugs should be manufactured using aseptic processing only when terminal sterilization is not feasible.”	“This document is intended to provide guidance for the submission of information and data in support of the efficacy of sterilization process in drug applications for both human and veterinary drugs.” “Regardless of whether the applicant uses terminal sterilization or aseptic processing to manufacture a drug product that is purported to be sterile, certain information about the validation of that process should be submitted for both types of sterilization.”

SAL, sterility assurance level; N/A, not applicable.

### European medicines agency

**FIG. 1.**
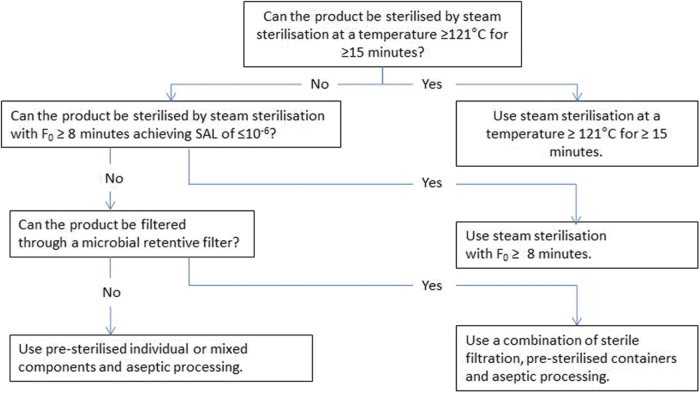
EMA decision tree for sterilization choices for aqueous products. EMA, European Medicines Agency.

The EMA guideline from 2019: “Guideline on the sterilisation of the medicinal product, active substance, excipient and primary container” [9] provides the most direction for selecting a sterilization method, which for aqueous products includes steam sterilization, sterile filtration coupled with aseptic processing, or aseptic processing of presterilized components (see EMA Decision Tree, Fig. 1) [4]. As with previous guidelines, the expectation that TS is the primary choice for processing is reiterated. The EMA concludes that sterile filtration and aseptic processing methods are vulnerable to the risk of accidental contamination and this risk cannot be eliminated by monitoring and control. Under the guideline, implementation of TS should be done “whenever possible,” and a robust justification must be provided when other sterilization methods are used. However, what constitutes a robust justification that sufficiently precludes the use of TS is open to varying interpretation.

Additional criteria in the EMA guidance are provided to aid in understanding the regulators' perspective on what does or does not preclude TS for a given product. For instance, according to the EMA, TS should not be ruled out based on an increase in degradation products or based on a reduction in shelf life. The guidance also states that substantial efforts, such as adjustments in pH, excipients, container, manufacturing conditions, and optimization of the sterilization method, should be explored to enable TS. Ultimately, an assessment of the risk to the patient of increased impurities versus the potential of releasing nonsterile units following aseptic processing needs to be made.

Other specific recommendations made in the EMA guideline that are generally more prescriptive than prior guidance are as follows:

● A numerical limit for bioburden concentration (100 cfu/100 mL) before TS.
● Recommendations for TS cycle development are made: cycles defined by the European Pharmacopoeia (Ph. Eur. 5.1.1), which requires products to be heated at a minimum of 121°C for at least 15 min, should be used when possible. Minimum cycle criteria are also provided, such as the minimum cycle lethality (*F*_0_ ≥ 8 min), minimum sterilization hold temperature (110°C), and sterility assurance level (SAL ≤10^−6^).
● Expectations for validation, including characteristics of the biological indicator, that is, D_121_ ≥1.5 min, are provided.
● Validation data to be included in the dossier, depending on the parameters of the sterilization cycle, process controls, and level of routine bioburden characterization, are provided.
● Proposal for less lethal cycles (*F*_0_ < 8 min), termed “terminal heat treatment,” to be used for the mitigation of inadvertent contamination during aseptic processing.

Of the specific recommendations made in the EMA guidance, two are significant sources of uncertainty for those beginning to evaluate and develop sterilization processes. These are the requirements regarding F_0_ cycles and relation to SAL, and the use of terminal heat treatment following aseptic processing. It is recognized by the authors that a SAL ≤10^−6^ can be readily achieved by processes with a lethality of *F*_0_ < 8 min when bioburden concentration is initially low or known to have a small D_121_ value. However, the EMA is explicitly recommending both criteria for the cycle lethality and sterility assurance are met. Therefore, the authors recommend that these criteria are considered and applied appropriately during feasibility and process development. More details regarding cycle development are provided in later sections of the article.

Regarding terminal heat treatment, which by the EMA definition is not considered a sterilization process, the authors do not believe its implementation is warranted. Our view is in line with the Parenteral Drug Association [9], who support inclusion of both aseptic and TS approaches in the guidance, but believe that there is a lack of scientific and risk-based evidence to support additional heat treatment for products that are manufactured under well designed and properly controlled aseptic conditions. Developing the requirements for such a cycle and justifying its use may also rely on mostly arbitrary information and hypothetical risk scenarios rather than known process capabilities and safety requirements. Therefore, the authors recommend following the decision tree provided in the guidance, which does not include the option of terminal heat treatment, when selecting the sterilization method.

Overall, the authors welcome the new EMA guidance and believe it provides greater insight into the regulator's expectations than previous guidance on that topic. Particularly helpful are the information found in the decision trees and requirements for cycle development and validation. However, even with this enhanced direction, it is acknowledged that the assessment of which processing method to select will be nuanced and will require numerous assumptions regarding the product, process capabilities, and rely heavily on the judgment of individual subject matter experts.

Furthermore, the lack of clear benchmarks for critical aspects like degradation and impurity levels, the need to make decisions relatively early in development, especially for accelerated programs, and the large downstream impact of decisions on late-stage process development timelines increase the stakes for manufacturers. Therefore, we recommend sponsors engage with regulators early and often to ensure alignment on the risks and technical merits of the processing strategy. In the following sections, we also aim to provide recommendations for a general development paradigm, and more specific technical recommendations to complement the regulatory guidance.

## General Development Paradigm

**FIG. 2.**
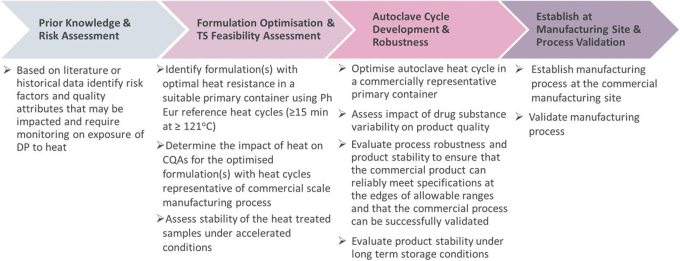
Terminal sterilization development paradigm for aqueous oligonucleotide products.

The development of a TS process for oligonucleotide drug products should follow a risk and science-based approach consistent with ICH Q8 and ICH Q9. Due to the vast array of oligonucleotide chemistries with different thermal characteristics and stability, there is no one-size-fits-all approach for evaluating and implementing autoclave sterilization. Each program will require its own assessment of formulation, container, and process conditions. However, conserved characteristics within some oligonucleotide subclasses may allow science-based generalizations at the start of development (chevron 1, Fig. 2) to reduce the overall development time.

For instance, early theoretical risk assessment may identify vulnerabilities that could preclude TS and be easily confirmed in the laboratory or with existing data. This may apply to oligonucleotides conjugated to heat-labile moieties such as proteins, double-stranded oligonucleotides with relatively low melting temperatures (eg, <110°C), or aptamers, which must maintain a high-order structure. For molecules that are more thermally stable, such as some ASOs, certain generalizations regarding the optimal pH of the solution, formulation composition, and profile of the autoclave cycle can be made to help focus subsequent formulation optimization (chevron 2, Fig. 2) and autoclave cycle development (chevron 3, Fig. 2). These generalizations and recommendations are discussed in the following sections.

When establishing the manufacturing process at the commercial site and validating the process (chevron 4, Fig. 2), many of the related activities will require to be repeated for each program. However, prior knowledge from other programs and modalities should be leveraged where appropriate, such as general equipment characterization studies, for example, autoclave cycle heat mapping.

Due to the significant amount of time required to develop, characterize, and validate a robust TS process for oligonucleotides, the authors believe the most appropriate entry point for implementing a TS process is during commercial process development. At this stage, the proposed commercial presentation and manufacturing site intended for launch have been selected, which is critical for the later stages of process characterization and validation where significant at-scale work is required. Ideally, the terminally sterilized product would be supplied to support pivotal clinical studies, but introduction before marketing authorization or as part of life cycle management may also be pursued if justified and with an appropriate comparability strategy.

## Recommendations for Formulation Development to Enable Autoclave Sterilization of ASO Drug Products

### Oligonucleotide degradation overview

**FIG. 3.**
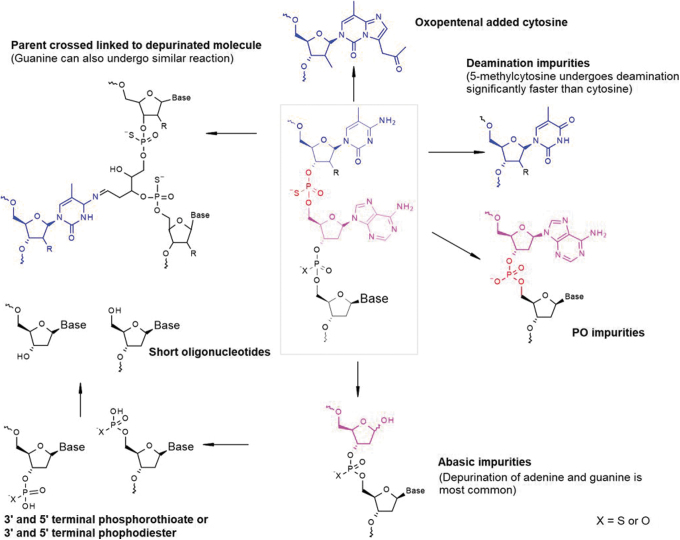
Common degradation products for antisense oligonucleotides.

Several comprehensive reviews are available, which discuss in detail the degradation pathways of oligonucleotides, but a brief overview is warranted in the context of potential degradation products created by thermal stress during TS [10,11]. The stability profile and nature of the degradation products produced at high temperature are dependent on the oligonucleotide chemistry, sequence, drug product formulation, excipients, manufacturing process, and storage conditions. Although one should exercise caution against sweeping generalizations, a number of chemical features and associated degradation pathways are common among many oligonucleotides, and the degradants formed during TS are shared with typical thermal stress studies. These pathways merit a discussion as they can inform the product quality monitoring strategy during TS development. Figure 3 illustrates degradation pathways for a single-stranded oligonucleotide with a phosphorothioate backbone, and ribose- and deoxyribose-based sugars, features that are ubiquitous to many therapeutic oligonucleotides.

Depurination, the release of purine bases by the hydrolysis of N-glycosidic bonds, is one of the most common degradation pathways for ASOs. The resulting degradation products containing apurinic sites are called abasic impurities. Depurination is primarily an acid-catalyzed hydrolysis where the rate is inversely correlated with the ionic strength and pH and has been shown to follow first-order kinetics [12,13]. Abasic impurities also readily undergo secondary degradation to form 3′ and 5′ shortmers with or without a terminal phosphorothioate or phosphate group. Depurination rate increases significantly at an elevated temperature and therefore abasic impurities and their secondary degradation products, for example, shortmers, oxopentanal cytosine [14], and cross-linked impurities, should be closely monitored during TS assessment of oligonucleotides. An additional concern regarding depurination is the potential for subvisible and visible particulate formation due to precipitation of free purines released in solution.

Guanine has a very low solubility of 3.8 μg/mL in aqueous solutions at the physiological pH range typically used for oligonucleotide formulations [15] and may precipitate in case of significant depurination during TS, or during the shelf life of the product. Although it is the author's experience that the presence of oligonucleotide may slightly increase the solubility of free guanine in solution, such precipitation events following significant thermal stress have been observed. The stochastic nature of precipitation events should also be considered when developing studies and evaluating data; therefore, the concentration and thermodynamic solubility of purines in the drug product are more robust indicators of the risk for precipitation than visual assessment for particulates.

Another degradation pathway to consider is the deamination of cytosine and 5-methylcytosine bases. These bases undergo hydrolytic deamination to yield uracil and thymine residues, respectively. The rate of deamination for 5-methylcytosine bases is roughly five times faster than cytosine [16]. Adenine also undergoes deamination, but at a significantly slower rate than cytosine, and therefore is not generally a degradation product of concern [17]. The deamination rate of 5-methylcytosine is lowest around physiological pH conditions and increases rapidly for more acidic and basic formulations [18].

Activation energy for the deamination of 5-methylcytosine residues in a single-strand oligonucleotide is reported to be ∼25 kcal/mol, suggesting deamination is a degradant of concern at elevated temperatures [16,19]. Due to their structural similarity, it is challenging to chromatographically resolve deaminated impurities from the parent oligonucleotide. In addition, because deamination results in change of molecular mass by 1 Da and only minor shift in isotopic distribution pattern, advanced mass spectrometric methods or other advanced methods with custom reagents are required to quantify these impurities with precision [19,20].

Phosphorothioate diester oligonucleotides may also undergo hydrolytic or oxidative desulfurization to form phosphate diester impurities. These pathways are accelerated at the elevated temperatures experienced during heat sterilization [21–23].

In summary, understanding degradation pathways, reaction kinetics, and factors that influence the rate of degradation is important for developing more heat-resistant formulations. Forced degradation data or information derived from literature can provide valuable information to identify vulnerabilities and aid the development of drug product formulation and TS cycle, as well as the selection of appropriate analytical methods.

### Formulation considerations

To ensure maximal shelf life and physiological compatibility, oligonucleotides are typically formulated in a pH range of 7.0–8.5. Because depurination and deamination are pH dependent and are often leading degradation mechanisms [18,24], and the impact of pH on the degradation rate is more pronounced as the temperature increases, the selection of the formulation pH is critical for limiting degradation during the TS process. The impact of pH on oligonucleotide stability may also vary based on sequence and particular vulnerabilities of the molecule; therefore, careful consideration must be made to select the optimal pH for the product.

The buffer system used to control pH should also be carefully considered. A primary concern is the ability of the buffer system to maintain a stable pH at high temperature during the TS process as well as over the shelf life of the formulation. Buffers with a large temperature coefficient, such as tris(hydroxymethyl)aminomethane (Tris), are not desirable as pH drift during sterilization will expose the product to significantly lower pH than targeted. As previously mentioned, the combination of low pH and heat exposure will increase degradation, even over the relatively short duration of an autoclave cycle.

Since oligonucleotides are typically formulated near physiological pH, there are relatively few pharmaceutically relevant buffers to choose from those that effectively buffer in the 7.0–8.5 range (Table 3). Any chosen buffer must also be compatible with the oligonucleotide and container closure system, ideally be an approved compendial excipient, and have a low temperature coefficient. Based on these considerations, a phosphate buffer system provides the best opportunity for enabling TS. The authors recommend evaluating phosphate-buffered formulations during development, provided there is no precluding factor related to the quality target product profile or safety.

**Table 3.** List of Common Pharmaceutically Relevant Buffers with Buffer Capacity in the pH 7.0–8.5 Range and Considerations for Their Use in Formulations for Terminally Sterilized Antisense Oligonucleotides

Buffer	pKa (25°C) [32]	Buffer range^a^	Temperature coefficient (dpKa/dT or dpH/dT)	Predicted pH at 121°C^b^	Comments
Histidine (pK_2_)	6.00	5.0–7.0	−0.022 [33]	5.3	Buffering range at the edge of desired formulation pH range. Large temperature coefficient.
Carbonate (pK_1_)	6.38	5.3–7.3	ND^c^	ND^c^	Buffering range at the edge of desired formulation pH range. Low temperature coefficient. CO_2_ solubility decreases with increasing temperature.
Citrate (pK_3_)	6.40	5.4–7.4	0.003 [34]	7.7	Buffering range at the edge of desired formulation pH range. Citrate buffer associated with increased pain on injection [ 25].
Phosphate (pK_2_)	7.21	6.2–8.2	Negligible [34]	7.4	Ideal buffering range. Negligible temperature coefficient. Compatible with oligonucleotides.
Tris	8.08	7.0–9.0	−0.018 [34]	5.7	Compatibility issue with oligonucleotides. Large temperature coefficient.

^a^ Assuming sufficient buffering capacity ±1 pH unit from the pK_a._

^b^ Assuming the temperature coefficient is linear across the temperature range of 25°C–121°C and a starting pH of 7.4 at 25°C.

^c^ Not determined.

The buffering capacity of the formulation is also an important factor that should be explored during development. Oligonucleotides formulated in water have inherent buffering capacity and at high drug concentrations (150–200 mg/mL), the solutions are typically isotonic or hypertonic. Because of this, buffer salts are often omitted to ensure physiological compatibility for high-concentration oligonucleotide formulations. For lower concentration formulations, a buffer is often required to ensure proper pH control during manufacturing and shelf life.

**FIG. 4.**
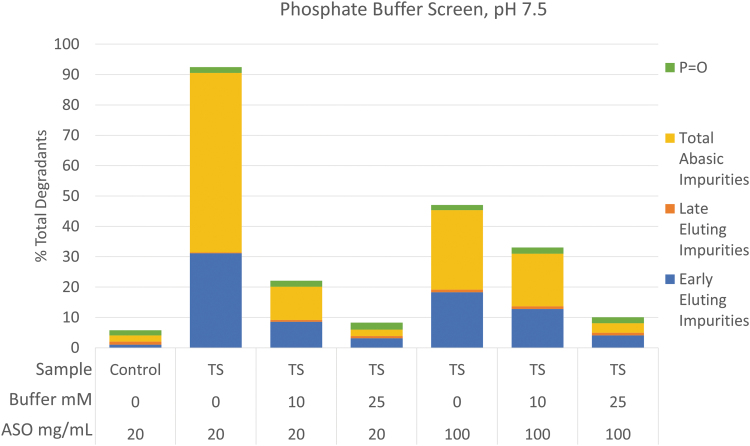
The effect of formulation buffer on the stability of a model oligonucleotide during terminal sterilization at 115°C for 15 min.

The effect that buffering capacity can have on the stability of a single-stranded 3-10-3 gapmer oligonucleotide drug product during TS is shown in Fig. 4. The oligonucleotide in this previously unpublished data is a relatively unstable sequence used for illustrative purposes, so this sort of extreme instability toward heat is not typical of most oligonucleotides. However, it does highlight some conserved trends observed across other oligonucleotide sequences regarding buffering capacity. For instance, in this example, drug products formulated at 20 and 100 mg/mL without added buffer show significantly different stabilities, with the 100 mg/mL solution showing about half the degradation due to the inherent buffering capacity of the oligonucleotide.

While the oligonucleotide does possess inherent buffer capacity, it has been noted during studies performed by several of the authors that, in general, as the oligonucleotide concentration is increased, more buffering capacity is needed to achieve optimal stability. For instance, the 20 mg/mL formulation in 10 mM phosphate buffer shows significantly less degradation than the 100 mg/mL formulation with the same amount of added buffer, and much less differentiation between formulations is seen as the buffer concentration is increased to 25 mM. It is hypothesized that the addition of phosphate buffer helps to minimize negative pH drift of the oligonucleotide solution during sterilization, which is driven by a drop in apparent pKa of the oligonucleotide at high temperature, and that at higher ASO concentrations, more phosphate buffer capacity is required to stabilize the pH. Generally, 10–25 mM of buffer is sufficient to achieve optimal stability.

## Product Quality Aspects of ASOs Post-TS

### Acceptable impurity levels following TS

Determining the nature and levels of individual degradation products and extent of total degradation upon exposure to a TS cycle forms a critical part of the development data package required to demonstrate suitability of the sterilization process. For small molecules, ICH Q3A “Impurities in New Drug Substances,” ICH Q3B “Impurities in New Drug Products,” and ICH M7 “Assessment and Control of DNA Reactive (Mutagenic) Impurities in Pharmaceuticals to Limit Potential Carcinogenic Risk” provide a regulatory framework that can be used to assess whether individual impurity levels have exceeded the recommended safety thresholds. Formation of degradation products above these thresholds, unless covered by the toxicological studies, provides a justification for sterile filtration and aseptic processing.

As mentioned previously, synthetic oligonucleotides are excluded from ICH Q3A and ICH Q3B; therefore, the impurity thresholds proposed within do not apply. In this regard, a position article published by Capaldi *et al.* [7] provides scientific advice on the control and qualification of product-related impurities in oligonucleotide therapeutics. For product-related impurities, the white paper proposes identification and qualification thresholds of 1.0% and 1.5%, respectively. The proposed control strategy for impurities is widely used in the industry for therapeutic oligonucleotides and has been accepted for a number of approved drugs. We believe that the proposed identification and qualification thresholds are also applicable to terminally sterilized products as considerations for patient safety remain the same.

In addition to controlling the individual impurities in the drug product, the increase in total degradation products on exposure to heat during steam sterilization should also be minimized. A significant increase in total degradation products not only poses a greater safety risk to the patients but also has potential to impact key product quality attributes such as appearance, assay, visible and subvisible particles, and pH.

With specific consideration of assay, we believe a significant increase in total degradation products and a commensurate reduction of drug product assay will significantly impact product quality and process robustness. For example, an assay specification limit of 95%–105% is considered appropriate for release in line with EMA guideline 3AQ11a “Specification and Control Tests on the Finished Product.” Considering inherent drug product assay variability due to the manufacturing process (compounding and dilution) and analytical variability, the assay loss caused by degradation during TS increases the risk of the drug product failing the lower assay release specification limit of 95%.

**FIG. 5.**
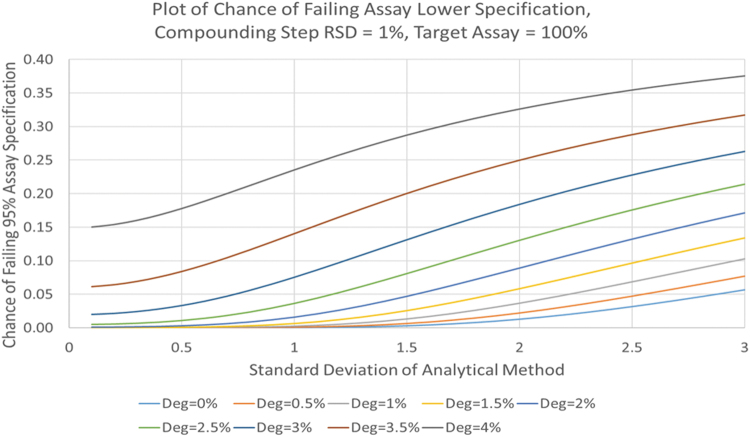
Probability of drug product failing the lower assay limit of 95% due to Degradation (Deg) during Terminal Sterilization (RSD = Relative Standard Deviation).

Figure 5 shows the impact that various degrees of degradation have on the probability of a batch failing the 95% assay release specification limit under a range of analytical variabilities. Under typical conditions, where analytical assay variability is in the order of 1%–1.5%, about 5%–15% of batches are expected to fail the lower release limit as degradation reaches 3%. The addition of an API overage can compensate for assay losses caused by TS, but in general is not recommended, as this would not avoid the increased level of degradation products, increased physical stability risks (eg, particulate formation), and is also discouraged in ICH Q8 (R2).

Drug substance quality is another critical factor that should be considered when assessing whether TS is an appropriate method of sterilizing the drug product. Due to the structural similarity of product-related impurities to the parent, the opportunity to remove impurities generated during synthesis is limited, and therefore, typical oligonucleotide drug substances have significantly higher level of impurities than small molecule drugs. Drug substances with different levels and types of impurities may lead to variability in drug product quality when exposed to heat during TS. For instance, a DS batch with a relatively large amount of depurinated impurities will likely result in a drug product lot with a disproportionately large amount of shortmer impurities, which may pose a risk to meeting specification. To ensure that commercial product consistently meets the required quality standards, TS studies using drug substance batches at the edges of allowable ranges should be considered.

## Recommendations for the Development of Autoclave Sterilization Processes for ASO Drug Products

### Assessing the feasibility of moist heat sterilization

The EMA guidance states that efforts made to implement TS should be presented in the regulatory filings. The information may include, but is not limited to, risk assessments, benefit-risk analyses, and development data. As described in Fig. 2, if prior knowledge and risk assessments indicate TS may be feasible for a particular product, development studies should be performed.

These studies should aim to provide sufficient information to draw conclusions on the feasibility of TS, and if possible, identify optimal formulations, autoclave cycles, and establish compatibility of container systems. The studies should be carried out using the most heat-stable formulation(s) available, bracketing oligonucleotide concentrations if variable dosing or dose is uncertain, and using primary containers constructed of materials representative of those anticipated for the commercial presentation. For instance, a glass vial may be a suitable surrogate for a glass PFS at this stage of development, but should always be supported with appropriate justification.

The autoclave cycle used during early development should be designed to meet the minimum requirements of the EMA guideline for sterilization, that is, *F*_0_ ≥ 8 min, with a minimum process hold temperature of 110°C. However, it is advised that the sterilization cycle validation, microbial control strategy, and equipment capabilities are also considered when determining a minimum cycle that is practical for future application. Without proper consideration of the viability of the cycle for commercial GMP manufacturing, feasibility studies might be performed under nonrepresentative conditions and may overestimate the robustness of the product. We have developed general recommendations regarding the lethality of cycles used in early development based on these considerations.

First, we must anticipate microbial validation requirements. The EMA guideline recommends that biological indicators used for biological validation have a decimal decay value of at least 1.5 min at 121°C (D_121_ ≥1.5 min), which indicates the time at 121°C to achieve a 1 log reduction in the microbial concentration [Ph. Eur. 5.1.2]. The guidance also states that the microbial control strategy should control the initial bioburden concentration below 100 CFU per 100 mL for a product stream to be suitable for TS. Because container size and fill volumes can vary greatly between products, and bioburden is often inhomogeneous in the bulk solution, it is reasonable to assume that any filled unit may contain up to 100 CFU.

With these design inputs, a cycle lethality of *F*_0_ ≥ 12 min is required to achieve an SAL ≤10^−6^ (8 log reduction). Therefore, a cycle of *F*_0_ = 12 min is a more practical minimum cycle to use during feasibility studies than the minimum cycle of *F*_0_ ≥ 8 min referenced in the EMA guidance. Assuming a lower D-value and starting bioburden concentration during feasibility studies, which would allow for a reduced cycle lethality, is not recommended, as doing so would require advanced knowledge of the microbes present at the future manufacturing site and, per the EMA guidance, require routine microbial characterization during processing. Such information is not often available at this stage and could change over the course of development.

Another reason for pursuing a cycle during development that is more lethal than *F*_0_ = 8 min is that the small-scale or pilot equipment available may have different characteristics and capabilities from the commercial-scale autoclaves. For instance, heating and cooling rates may be significantly different. Degradation occurring during the temperature ramp-up and ramp-down, where the temperature is below the minimum sterilization temperature (110°C), can result in significant degradation with no additional accumulation of cycle lethality.

Minimizing these times is important for ensuring the success of the commercial process, but doing so during early development may result in the generation of data at cycle conditions that cannot be successfully transferred. Therefore, the goal is not to minimize heating and cooling times, but to mimic as best as possible the anticipated capability of the commercial equipment. This will allow the development studies to be as representative as possible and provide the best information for making decisions regarding the feasibility of TS.

The examples above assume F_0_ control of the equipment; however, autoclave cycles are more commonly controlled by setting the sterilization hold temperature and hold time. When performing early development, these cycles should meet the minimum requirements of the EMA guidance, and therefore should have a temperature ≥121°C for ≥8 min (ie, *F*_0_ ≥ 8 min). However, to ensure a feasibility experiment is representative of the potential heat exposure of the commercial process; it is necessary to include the tolerances expected in the commercial-scale equipment. Considering a typical autoclave sterilization temperature band of 2°C, it is recommended that early development studies controlled by temperature and time should have a sterilization temperature at the top of the band, for example, 123°C, and hold time of 8 min (Table 4).

**Table 4.** Recommended Sterilization Conditions During Feasibility and Early Development Studies

Autoclave cycle control	Target sterilization temperature (°C)	Target hold time of sterilization phase (min)	Target or calculated cycle lethality (F_0_)	Calculated Log reduction^a^	Sterility assurance level (SAL)^d^
F_0_	121	N/A	12.0^b^	8 log	≤10^−6^
Temperature and time	123	8	12.7^c^	8 log	≤10^−6^

^a^ Assuming D_121_ = 1.5 min.

^b^ Sterilization credit should be accumulated at temperatures ≥110°C during heat-up and hold phases only.

^c^ Assuming Z-value of 10°C. Does not include additional lethality occurring during heating or cooling.

^d^ Assuming maximum microbial load of ≤100 CFU per unit during filing and bacteriostatic growth conditions (eg, 5°C storage) from end of filling to start of sterilization.

N/A, not applicable.

The authors do not recommend exploring sterilization temperatures below the reference temperature of 121°C during development, although the minimum sterilization temperature per the guidance is 110°C. This is because for most drug substances, including ASOs, lower sterilization temperatures (<121°C) are not expected to result in less degradation for the same process lethality. This conclusion is derived from fundamental characteristics of microbial death curves and chemical degradation. First, there is the z-value, which is the change in temperature required to alter the D-value (decimal reduction value, ie, the duration required to reduce the number of viable microorganisms to 10% of the original value) by a factor of 10. It is broadly accepted that this value is assumed to be 10°C during development of sterilization cycles, meaning that for a reduction of the sterilization temperature by 10°C, a 10 times increase in sterilization time is required to achieve the same lethality.

In contrast, the reduction in the degradation rate is much less significant for the same decrease in temperature. For instance, the rate constant for deamination, a first-order reaction mechanism with an activation energy of ∼25 kcal/mol [19], decreases only by a factor of 2.3 when the reaction temperature is decreased from 121°C to 110°C. Therefore, a reduction in temperature will result in more degradation for the same cycle lethality due to the longer hold time required during the sterilization phase. For this reason, it is not recommended to evaluate lower sterilization temperatures solely to limit degradation.

On the other hand, higher temperatures (>121°C plus sterilization temperature band) may limit degradation at equivalent lethality; however, realizing such benefit requires a higher level of process control for heating and cooling to minimize degradation during these phases, and the support pressure, particularly for PFS. And while such cycles may be feasible in development-scale equipment, the ability to adequately control and validate hotter cycles in commercial scale equipment needs to be considered before developing the process. Therefore, it is recommended to target a standard sterilization temperature of 121°C, plus applicable safety margins as discussed above, during initial feasibility studies.

### Autoclave cycle development and process validation

Once feasibility of TS for a given product is established, the next step is to optimize the cycle conditions using a commercially representative primary container and equipment. First, available commercial equipment in the manufacturing network needs to be identified to determine the cycle types and conditions to be explored. When defining and modifying cycle parameters, impact to SAL, product quality, product stability, and the container closure must be considered. In addition, process robustness should be evaluated by performing runs at the upper and lower temperature, time, and pressure ranges of the cycle.

For liquid products filled in closed containers, such as PFS, cartridges, and sealed bottles or vials, a support pressure must be supplied to minimize the differential pressure in the container. The two most common sterilization processes providing overpressure are the Steam-Air Mixture (SAM) and superheated water cycles. The support pressure applied in these systems helps maintain container shape and integrity, and for PFS and cartridges, prevent plunger-stopper movement. Addition of a drying step at the end of the cycle to remove residual water may also be considered if a dry load is required for the next stage in manufacturing.

Equipment capable of running these cycles are often not readily available in the development laboratory setting, and while it may be possible to conduct feasibility studies in less capable autoclaves, such as those used to sterilize laboratory equipment, there are safety concerns related to potential glass breakage or product spillage. Therefore, utilizing equipment capable of providing support pressure during early feasibility assessments is highly recommended, but using capable equipment during late-stage and commercial process development is essential for gaining a holistic understanding of product and container impacts.

Beyond choosing equipment with the right cycle capabilities, considerations of equipment performance and manufacturing scale should also be made. Depending on the equipment and facility specifications, the maximum sterilization temperature, support pressure, and achievable heating and cooling ramps can differ considerably from one sterilizer to another. For example, a pilot-scale autoclave used by an EPOC member for TS development allows for a maximum temperature of 125°C and pressure of 5.9 bar, whereas the commercial-scale autoclave considered for process transfer enables a maximum temperature of 135°C and a support pressure of 3.9 bar.

The impact of the packaging type, thermal mass of the load, and the scale of equipment on heating and cooling ramp rates should be considered during process development. Minimizing heat exposure during the nonsterilizing phases of the autoclave cycle helps to prevent excess thermal exposure of the product and degradation. For instance, parameter adjustments to optimize air removal and ensure rapid, uniform heating during the heating phase may help limiting unnecessary degradation of the product. The ability to match expected ramp rates in commercial equipment during development is also important for successful transfer of the process.

The ability to transfer and maintain control of critical parameter is the main consideration when scaling the sterilization process to the commercial site. Critical parameters are those that can directly impact the safety or efficacy of the product if not controlled within acceptable operating ranges. Included are parameters that directly impact the lethality of the sterilization process, such as exposure time, pressure, temperature, and F_0_. Parameters impacting the heating and cooling phases are typically not considered critical parameters for a TS process, but their impact on product quality should be carefully assessed and efforts made to optimize and control them during cycle development, as discussed above.

After transfer and characterization of the sterilization process at the commercial site are complete, the process must be validated according to the relevant regulations and guidance. For sterilization conducted according to the Ph. Eur. reference condition, validation is required, but the validation results do not need to be submitted in the marketing application. For less lethal cycles, physical and biological validation data from at least three sterilization runs must be provided [Ph. Eur. 5.1.1] [4]. In general, requirements for characterization, validation, and routine process controls increase as cycle lethality decreases [4].

## Considerations for Container Closure

The FDA Guidance for Industry: Container Closure Systems for Packaging Human Drugs and Biologics, defines the container closure system as “the sum of packaging components that together contain and protect the dosage form.” The container closure system should provide the necessary protection to the medication over its shelf life to minimize degradation occurring from “exposure to light, loss of solvent, exposure to reactive gases, for example, oxygen, absorption of water vapor, and microbial contamination.” When evaluating TS, it is important to ensure the primary container system retains its essential protective properties.

Glass is the most common primary packaging material for liquid pharmaceuticals, owing to it being chemically inert and compatible with most pharmaceutical formulation, and because it provides an impermeable barrier that prevents exchange of gases, solvents, and microbes. Glass is categorized by its chemistry and hydrolytic resistance. Hydrolytic resistance is ranked into three tiers, Type I–III. For liquid parenteral products, Type I, clear borosilicate glass is used almost exclusively. Pharmaceutical industry standards for glass containers are provided in Ph. Eur. 3.2.1 and USP <660>.

During development, the container closure system must be shown to be suitable for the drug product's intended use. This includes demonstrating the container closure provides the required level of protection to the product, is compatible with the product, is safe to use, and performs as expected. Ensuring the container closure is not negatively impacted by the manufacturing process, the product formulation, or storage conditions to the point at which it cannot serve its primary function or poses a risk to patient safety is an essential part of this effort.

The thermal and physical stresses experienced during steam TS pose several distinct risks to the container closure. Alteration of a container's surface chemistry, physical manifestations of chemical degradation, that is, delamination, release of leachable substances or lubricants, pressurization and its potential impact on plunger stopper movement, functionality (break-loose and gliding forces, needle pull-out force), or container integrity, are all aspects that may be adversely impacted by TS processes.

### Container closure integrity

Maintaining container closure integrity (CCI) during and following exposure to the intense heat and pressures experienced during steam sterilization should be carefully assessed. As discussed in the following paragraph, there is the potential for a pressure differential to arise between the container closure and the chamber during the TS process. While autoclaves used for sterilization of closed containers are equipped to supply support pressure, absolute control of the pressure differential cannot be expected.

Therefore, it is important to assess the potential impact that pressurization of the container may have on CCI, and it is recommended that qualification of CCI according to the relevant guidelines is performed with samples poststerilization for a given container system and fill weight. In addition to assessing CCI, ensuring residual seal force is sufficient and unchanged poststerilization for vial configurations may be assessed. For PFS, an assessment of plunger movement or disruption of tip cap placement is appropriate.

In general, PFS are more sensitive than vials to the pressure differentials experienced during heating due to the lack of an over seal and need to maintain the precise location of plunger stopper placement. An increase of vapour pressure and expansion of liquid in the head space may move the plunger of a syringe if the internal pressure is not adequately balanced with the external pressure, that is, the chamber pressure in the autoclave, and if the force generated by the pressure imbalance is larger than the break loose and gliding forces of the container closure system.

**FIG. 6.**
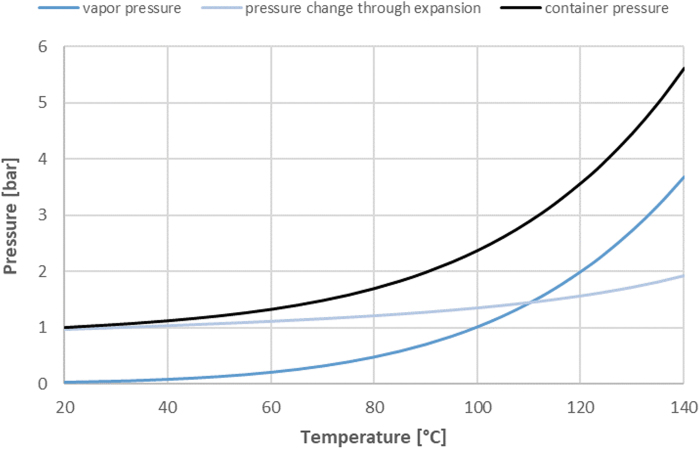
Internal container pressure of a 1 mL PFS filled with 0.65 mL fill volume and 0.1 mL head space as a function of temperature. PFS, prefilled syringes.

It is possible to calculate the movement of the plunger at different sterilization temperatures and support pressures. First, the container pressure can be calculated as described by Joyce and Lorenz [25]. As an example, the pressure change through expansion has been calculated for a 1 ml syringe with 0.65 mL fill volume and 0.1 mL head space (Fig. 6). In SAM autoclaves, the total pressure inside the chamber during the sterilization phase is given by the partial pressure of the steam (vapour pressure) and the partial pressure of the heated air (support pressure).

**FIG. 7.**
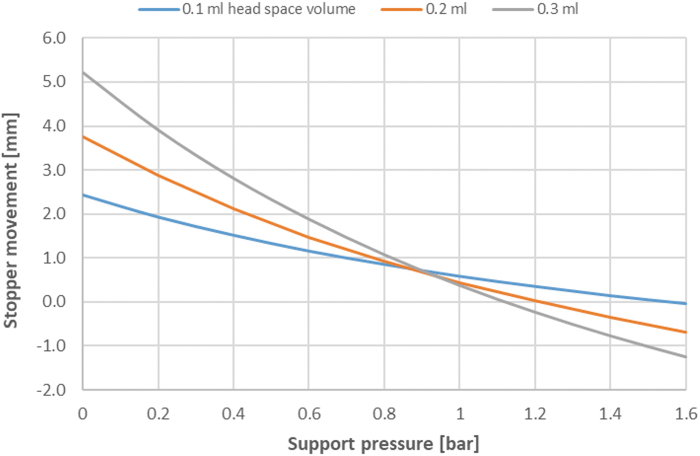
Worst-case theoretical plunger movement in a 1 mL PFS with 0.65 mL fill volume when sterilized at 121°C at varying support pressures. The different traces show the impact of having head space volumes of 0.1, 0.2, or 0.3 mL.

The plunger stopper movement resulting from the difference in the internal pressure and support pressure in the autoclave can be calculated using Boyle's law [26]. As an example, the plunger movement for a 1 mL syringe with 0.65 mL fill volume has been calculated for different support pressures, that is, pressures exceeding the vapour pressure at the sterilization temperature, and for different head space volume (Fig. 7). Note that the plots above are for illustration purposes and are considered worst case as the impact of frictional forces (break loose and gliding forces) has not been accounted for.

From Figure 7, it is seen that positive plunger movement (toward the syringe barrel flange) at low support pressure is greater in products with a larger headspace. It is also seen that movement can be minimized by selecting the appropriate support pressure during the sterilization phase. If the support pressure is too high, compression of the head space, resulting in a plunger movement toward the liquid, is also possible. Importantly, the support pressures should be adjusted as the temperature of the load changes. This is particularly critical during cooling, where the pressure from the vapour phase dissipates rapidly, while the load remains hot and under pressure.

There are several concerns regarding plunger stopper movement in syringes, such as liquid getting in the ribs of the plunger stopper or potential impacts on device assembly, secondary packaging, and device functionality. The most serious concerns are the potential for compromised container-closure integrity and sterility.

While limits for plunger stopper movement for a given container configuration should be assessed on a case-by-case basis, the distance must minimally limit migration of the product past the sterile boundary, which is typically equal to the distance between the first and last sealing ribs on the plunger stopper [26]. Even though the whole syringe is sterilized, the inside of the autoclave is not validated as a sterile area; therefore, movement beyond the sterile boundary established after placement during filling is not acceptable. Also, plunger stopper movement toward the syringe barrel flange, especially for a PFS filled close to nominal capacity, may put the plunger stopper closer to or within the flare-out zone, where the syringe barrel bore diameter increases. This condition may impact CCI or make the container more susceptible to plunger stopper movement during ambient pressure fluctuation, as may be expected during transportation.

### Compatibility and functionality

During product development, compatibility of the product formulation with the primary container must be established. Confirming minimal interaction between the product and container system includes ensuring no significant impact on product quality caused by the container system. Examples of this include product adsorption to surfaces or lubricants, or oxidation catalyzed by metals, such as residual tungsten left from the syringe barrel forming process. Ensuring the container system is compatible with the product formulation attributes, such as pH or ionic strength, is also critical. An example of this could be inadequate hydrolytic resistance to the formulation, resulting in damage of container surfaces and delamination.

Even when product formulation and container are sufficiently compatible, the thermal stresses experienced during moist heat sterilization may exacerbate otherwise innocuous incompatibilities into significant product quality risks. Therefore, the impact of the sterilization process must also be considered before choosing the final container system. This may include assessment of drug product quality attributes and the functionality of the container presterilization and poststerilization to ascertain if any change has occurred, or if the system has been rendered more susceptible to long-term storage stresses. For example, it is wise to evaluate the distribution of silicone oil after sterilization and check for the presence of silicone oil droplets in solution.

The disruption of silicone oil or other lubricant layers on the plunger stopper and syringe barrel during sterilization could potentially impact break-loose and gliding forces, or even the stability of the drug product due to increased surface area of lubricant dispersed as droplets in solution. Needle pull-out forces may be impacted by TS of PFS with staked needles due to a potential weakening of the adhesive. To increase confidence in container closure selection and compatibility with product, we recommend performing stability studies post-TS, and if possible, in comparison to product that has not been heat treated.

### Extractables and leachables

When selecting the primary packaging components for a drug product, it is critical to ensure the materials of construction do not leach hazardous levels of chemicals into the product. Extractable studies can help determine what compounds may be extracted, and at what levels. The studies are typically performed under harsh conditions using polar and nonpolar solvents, and pH extremes. Furthermore, extractable studies are often performed at elevated temperature, which can accelerate extraction. The Product Quality Research Institute (PQRI) [27,28] has established best practices for the justification, design, and implementation of extractable and leachable (E/L) studies of the container closure systems used in drug products, and the topic is also discussed in USP general chapters <1661>, <1663>, and <1664>, and the ICH Q3E concept article: “Guideline for Extractables and Leachables (E&L).”

Similar to extraction studies, the high temperature experienced during TS may accelerate the leaching of compounds into the drug product. In addition to assessing extractable data and determining the subset of extractables to monitor during long-term storage of the product, it is important to understand the impact of the TS process on the leachable profile. It is recommended that if leachable studies are required for a product, the TS process is incorporated into the study design, either through exposing samples to steam sterilization or other methods of modeling the thermal stress.

### Delamination

The type and quality of glass containers used for pharmaceutical products are defined in USP <660>/Ph. Eur. 3.2.1. Different types of glass are recommended for different intended uses, with Type 1 borosilicate glass recommended for liquid injectables due to its relatively high hydrolytic resistance. Nevertheless, weakening of the glass surface due to hydrolytic attack remains a potential quality concern for aqueous formulations stored in glass containers. The most serious manifestation of this phenomenon is glass delamination. Glass delamination occurs when the glass surface weakens to the extent that small fragments of glass lamellae slough off into the product formulation.

In 2011, FDA issued an advisory to manufacturers due to the elevated number of product recalls in previous years caused by glass delamination. The advisory recommended manufacturers assess products for potential risk of delamination and provided relevant risk factors. Such risk factors are now summarized in USP <1660>. Included are the type of container, container processing conditions, and factors related to the drug product, such as formulation pH, formulation composition, storage conditions, and shelf life, and whether the product is terminally sterilized.

High heat exposure during sterilization by autoclave has been shown to promote glass attack and increase chances of delamination [29]. To determine the potential impact to terminally sterilized oligonucleotide products, a study was performed by one of the EPOC member companies and presented here for the first time. In the study, Type 1 glass vials were filled with buffer according to the formulation recommendations described above (10 mM phosphate buffered saline, pH 7.7) and terminally sterilized. The sterilization condition evaluated was equivalent to the standard cycle of 121°C for 15 min (Ph.Eur. 5.1.1 reference condition) and all vials were subsequently stored at accelerated storage condition of 40°C for up to 20 weeks, simulating up to 5 years' storage at 5°C. A group of vials not sterilized were used as negative controls in the study.

Several analytical methods were used to assess surface durability immediately after TS and during storage, including visual inspection, stereomicroscopy, scanning electron microscopy with energy dispersive X-ray detection, and inductively couple plasma mass spectrometry and optical emission spectroscopy. While all terminally sterilized vials showed evidence of glass attack, glass attack was also observed for nonterminally sterilized vials stored for a simulated storage time of 2 years at 5°C or greater (Table 5). Early indicators of delamination were observed for some samples, but were not correlated to TS or storage time, and glass delamination was not observed for any sample.

**Table 5.** Delamination Risk Following Terminal Sterilization

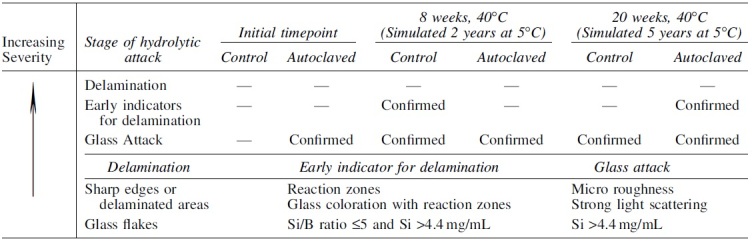

Even though delamination was not observed in the study, the observed glass attack and early indicators of delamination underscore the importance of understanding how product and process impact container durability. It is recommended that for each product, terminally sterilized or not, an assessment is made according to the risk factors in USP <1660>. If TS is used, it is recommended to increase the rigor of the assessment, and if necessary, perform similar studies aimed at investigating glass durability.

## Summary and Recommendations

Oligonucleotides constitute a heterogeneous group with fundamental differences in physical and chemical properties between subclasses. As such, EPOC advocates that the feasibility assessment for TS should be performed on a case-by-case basis and follow a scientific and risk-based approach, as described in Fig. 2. Throughout this evaluation process, the principles outlined in the decision tree provided in the EMA guidance should be followed.

Under certain circumstances, we believe that scientific and platform knowledge may be sufficient to rule out TS for a given chemical entity, provided there is deep understanding of the impacts of the sterilization process on the product, or other precluding factors related to the target product profile. For more thermally stable oligonucleotides and instances where deep product and process knowledge does not exist, feasibility must be assessed by well-designed and controlled laboratory studies. Several recommendations were made by the authors to guide developers through the early stage and late development of TS processes for oligonucleotides. These general recommendations and key takeaways, mostly specific to ASOs, are summarized in Table 6.

**Table 6.** General Recommendations for the Development of Terminal Sterilization Process for Oligonucleotide Drug Products

Focus topic	General recommendations
Recommendations for formulation development to enable autoclave sterilization of antisense oligonucleotides	
Excipients	Excipients should be chosen based on their ability to stabilize the active substance, provide a physiologically compatible delivery vehicle, withstand stresses of the sterilization cycle (eg, not degrade or precipitate), and be compatible with the container system.
pH	Formulation pH of 7.0–8.5 is optimal for maintaining stability during sterilization and is generally suitable for parenteral injection. Formulation pH lower than 7.0 and above 8.5 should be avoided.
Buffer type	Buffer should have a small temperature coefficient. Phosphate buffer is ideal for maintaining pH in the optimal pH range (7.0–8.0) before, during, and after sterilization.
Buffer capacity	Buffer capacity must be sufficient to minimize pH shift during sterilization. Generally, 10–25 mM phosphate buffer is sufficient for most oligonucleotide formulations.
Overages	Overages to compensate for the assay loss encountered during moist heat sterilization are not recommended and are discouraged in ICH Q8.
Stability	The stability drug product post-TS should be monitored at the desired long-term and accelerated storage conditions per ICH Q1A (R2). If data are available, it is recommended to compare degradation profiles to product that has not been moist heat sterilized.
Impurity type	The impurities created during moist heat sterilization are generally the same impurities generated under thermal stress conditions (ie, 40°C).
Impurity levels	If the level of an impurity increases during terminal sterilization beyond its toxicological qualification limit, then sterile filtration and aseptic processing may be justified. Significant changes to obligatory critical quality attributes such as appearance, visible and subvisible particulates, and pH may also preclude terminal sterilization, provided no other mitigation can be developed. An increase in degradation products as a result of TS is excessive if it risks failure of lower assay release limit (ie, 95%) when also considering analytical and manufacturing variability.

Recommendations for the development of autoclave sterilization processes for antisense oligonucleotides	
Method of sterilization	For aqueous oligonucleotide products, it is recommended to assess moist heat sterilization methods per the decision tree in the EMA guidance. It is not recommended to assess sterilization by irradiation or other chemical methods. Membrane filtration may be used if moist heat sterilization is not possible.
Feasibility assessments	If prior knowledge assessment indicates moist heat sterilization may be possible, feasibility studies should be performed. Feasibility studies should be carried out using the most heat-stable formulation(s) available and container closure with representative materials of construction. The goal of the study should be to generate enough data to confidently rule on the feasibility of terminal sterilization for the product.
Recommended sterilization cycles for feasibility assessments	The recommended moist heat sterilization cycle to use during feasibility studies is a cycle with an F_0_ of 12 min and target temperature of 121°C. Alternatively, if controlling by time and temperature, a cycle with a target sterilization temperature of 123°C and sterilization hold time of 8 min may be used.
Commercial sterilization cycle development	For cycles with the same overall lethality (F_0_), hotter, shorter cycles can minimize oligonucleotide degradation compared to cooler, longer cycles. Therefore, it is not recommended to use sterilization temperatures lower than 121°C. Cycles hotter than 121°C may help limit degradation, but may also require tighter equipment control and increased capabilities to be successfully implemented. Control of heating and cooling rates during development and process transfer must also be considered, as heat exposure during these steps may impact product quality.
Cycle validation	Validation of the cycle must be performed according to applicable regulations and guidance. Relevant information based on the type of cycle validated must be included in the marketing application dossier.

Container closure considerations	
Container closure integrity	The integrity of the proposed commercial container closure should be verified to ensure the TS process does not adversely impact the integrity of the system and ability to maintain sterility over the shelf life of the product.
Compatibility and functionality	Chemical and physical changes to the container during TS may significantly affect its suitability and functionality. Critical attributes of the container system should be assessed after sterilization and on stability.
Extractable and leachable compounds	The extreme conditions encountered during TS can increase the permeability of the container closure, especially the rubber plunger stopper, and increase the solubility of leachable compounds in the drug product. Assessments should consider these impacts, and if necessary, leachable studies that include exposure of the drug product or surrogate filled container closure to TS conditions should be performed.
Delamination	TS is a risk factor for glass delamination. A comprehensive risk assessment according to the concepts in USP <1660> should be conducted.
